# Tetra­kis(μ-3-aza­niumylbenzoato)-κ^3^ *O*:*O*,*O*′;κ^3^ *O*,*O*′:*O*;κ^4^ *O*:*O*′-bis­[tetra­aqua­neodymium(III)] hexa­chloride tetra­hydrate

**DOI:** 10.1107/S1600536811012700

**Published:** 2011-04-13

**Authors:** Meriem Benslimane, Hocine Merazig, Jean-Claude Daran

**Affiliations:** a Unité de Recherche de Chimie de l’Environnement et Moléculaire Structurale, Faculté des Sciences Exactes, Département de Chimie, Université Mentouri de Constantine, 25000 Constantine, Algeria; b Laboratoire de Chimie de Coordination, UPR-CNRS 8241, 205 route de Narbonne, 31077 Toulouse Cedex 4, France

## Abstract

The structure of the title compound, [Nd_2_(C_7_H_7_NO_2_)_4_(H_2_O)_8_]Cl_6_·4H_2_O, consists of dimeric cationic units related by an inversion centre. The two Nd^III^ atoms are linked by two bridging bidentate carboxyl­ate groups and two bidentate chelating bridging carboxyl­ate groups, with an Nd⋯Nd separation of 4.1259 (4) Å. Each Nd^III^ atom is nine-coordin­ated by five O atoms from the carboxyl­ate groups of the zwitterionic azaniumylbenzoate ligands and four from water mol­ecules. They adopt a distorted tricapped trigonal–prismatic arrangement. The dihedral angle between the mean planes of the benzene ring and the carboxlate groups are 7.7 (6) and 24.4 (5)°. The two carboxyl­ate groups are almost perpendicular to one another with a dihedral angle of 84.0 (7)°, while the two benzene rings are inclined to one another by 81.8 (2)°. The mol­ecular packing is stabilized by O—H_water_⋯Cl, O—H_water_⋯N, N—H⋯Cl, N—H⋯O, and O—H_water_⋯O hydrogen bonds and π–π stacking inter­actions [centroid–centroid distance = 3.500 (3) Å] between symmetry-related benzene rings. All of the Cl^−^ anions and the uncoordinated water molecules are disordered over two sets of sites with different occupancy ratios.

## Related literature

For applications of lanthanide complexes, see: Yan *et al.* (1997 ▶); Scott & Horrocks (1992 ▶). For lanthanide complexes with aromatic carb­oxy­lic acids, see: Ma *et al.* (1994 ▶). For similar complexes, see: Qin *et al.* (2005 ▶, 2006 ▶); Sun *et al.* (2002 ▶); Benslimane *et al.* (2011 ▶).
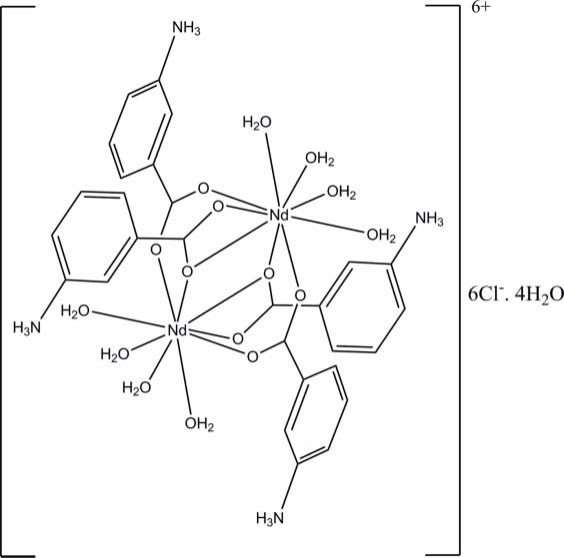


## Experimental

### 

#### Crystal data

[Nd_2_(C_7_H_7_NO_2_)_4_(H_2_O)_8_]Cl_6_·4H_2_O
*M* *_r_* = 1265.92
Monoclinic, 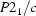

*a* = 12.1717 (1) Å
*b* = 19.8544 (1) Å
*c* = 10.5170 (1) Å
β = 112.018 (1)°
*V* = 2356.19 (4) Å^3^
*Z* = 2
Mo *K*α radiation
μ = 2.59 mm^−1^
*T* = 293 K
0.30 × 0.24 × 0.16 mm

#### Data collection

Enraf–Nonius CAD-4 diffractometer
Absorption correction: multi-scan (Blessing, 1997 ▶) *T*_min_ = 0.410, *T*_max_ = 0.444
7192 measured reflections
6852 independent reflections
4724 reflections with *I* > 2σ(*I*)
*R*_int_ = 0.031
2 standard reflections every 60 min  intensity decay: 3%

#### Refinement

*R*[*F* ^2^ > 2σ(*F* ^2^)] = 0.041
*wR*(*F* ^2^) = 0.096
*S* = 1.02
6852 reflections
300 parameters
H-atom parameters constrained
Δρ_max_ = 0.81 e Å^−3^
Δρ_min_ = −1.15 e Å^−3^

### 

Data collection: *CAD-4 EXPRESS* (Enraf–Nonius, 1994 ▶); cell refinement: *CAD-4 EXPRESS*; data reduction: *XCAD4* (Harms & Wocadlo, 1996 ▶); program(s) used to solve structure: *SIR92* (Altomare *et al.*, 1993 ▶); program(s) used to refine structure: *SHELXL97* (Sheldrick, 2008 ▶); molecular graphics: *ORTEPIII* (Burnett & Johnson, 1996 ▶) and *ORTEP-3 for Windows* (Farrugia, 1997 ▶); software used to prepare material for publication: *SHELXL97*.

## Supplementary Material

Crystal structure: contains datablocks I, global. DOI: 10.1107/S1600536811012700/su2265sup1.cif

Structure factors: contains datablocks I. DOI: 10.1107/S1600536811012700/su2265Isup2.hkl

Additional supplementary materials:  crystallographic information; 3D view; checkCIF report

## Figures and Tables

**Table 1:** Hydrogen-bond geometry (Å, °)

*D*—H⋯*A*	*D*—H	H⋯*A*	*D*⋯*A*	*D*—H⋯*A*
N1—H1*A*⋯O6*WA*	0.89	2.16	3.007 (12)	160
N1—H1*B*⋯Cl1	0.89	2.30	3.174 (5)	168
N1—H1*C*⋯O6*WA*^i^	0.89	1.98	2.828 (12)	159
N2—H2*A*⋯O4^ii^	0.89	2.22	2.967 (6)	141
N2—H2*B*⋯Cl2^iii^	0.89	2.31	3.166 (5)	161
N2—H2*C*⋯Cl3*A*^iv^	0.89	2.26	3.129 (5)	167
O1*W*—H11⋯Cl2^v^	0.97	2.21	3.170 (4)	172
O2*W*—H12⋯O5*W*	1.04	2.28	2.960 (7)	121
O2*W*—H12⋯Cl2^v^	1.04	2.65	3.443 (5)	132
O3*W*—H13⋯Cl2	0.95	2.26	3.190 (5)	169
O4*W*—H14⋯Cl3*A*^vi^	0.86	2.58	3.240 (5)	134
O5*W*—H15*W*⋯Cl1^vii^	0.85	2.68	3.208 (7)	122
O1*W*—H21⋯Cl1^iv^	0.78	2.52	3.215 (4)	148
O2*W*—H22⋯Cl2	0.90	2.26	3.104 (4)	156
O3*W*—H23⋯Cl3*A*	0.80	2.56	3.244 (4)	144
O4*W*—H24⋯Cl1^viii^	0.93	2.23	3.134 (5)	163
O5*W*—H25*W*⋯N1^iii^	0.85	2.52	3.247 (8)	144
C10—H01⋯Cl1^iv^	0.93	2.81	3.724 (6)	169
C14—H04⋯O2*W*^iii^	0.93	2.59	3.504 (7)	170

Symmetry codes: (i) 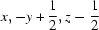; (ii) 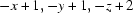; (iii) 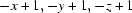; (iv) 
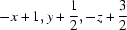
; (v) 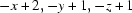; (vi) 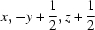; (vii) 
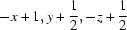
; (viii) 
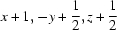
.

**Fig. 1.**
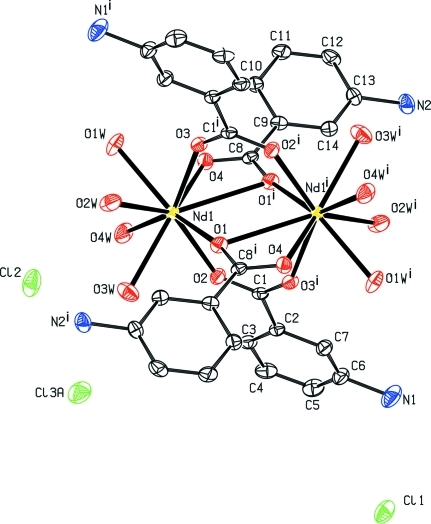
The molecular structure of the title compound, with the atom-numbering scheme. Displacement ellipsoids are drawn at the 30% probability level [Symmetry code: (i) -x + 1, -y + 1, -z + 1; Hydrogen atoms have been omitted for clarity].

**Fig. 2.**
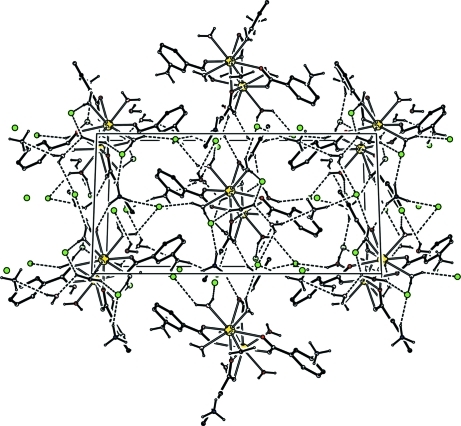
The crystal packing of the title compound, viewed approximately down the a axis. Hydrogen bonds are shown as dashed lines [see Table 1 for details; hydrogen atoms not involved in hydrogen bonding have been omitted for clarity].

## supplementary crystallographic information

### Comment

In recent years, much research has been done on lanthanide coordination compounds with some organic ligands, which have chelated structures and exhibit photophysical properties for the application in luminescence probes for chemical or biological macromolecules and the active center for molecular based luminescent materials (Yan *et al.*, 1997; Scott *et al.*, 1992). Especially lanthanide complexes with aromatic carboxylic acids show higher thermal and luminescent stability for practical applications than other lanthanide complexes because they readily form dimer or infinite chain polymeric structures (Ma *et al.*, 1994).We report herein on the preparation and crystal structure of the title compound.

The molecular structure of the title compound consists of dimeric units related by an inversion centre (Fig. 1). The two Nd^III^ atoms are linked by two bridging bidentate carboxylate groups and two bidentate chelating bridging carboxylate groups. Each Nd^III^ atom is nine-coordinated by five O atoms from carboxylate groups of the 3-ammoniumbenzoate, and four O atoms from the water molecules. They adopt a distorted tricapped trigonal-prismatic arrangement. A similar coordination environment was observed previously for lanthanoid(III) complexes, such as [(pyridine-3,4-dicarboxylate)_2_(NO_3_)_2_(H_2_O)_3_] (Qin *et al.*, 2006) and [Ln_2_(imidazole-4,5-dicarboxylate)_2_(H_2_O)_3_]1.5H_2_O (Ln = Sm and Eu; Qin *et al.*, 2005). The Nd-O distances involving the carboxylate groups range from 2.394 (3) Å to 2.458 (4) Å and those of the Nd-Owater bonds from 2.506 (4)Å to 2.525 (4) Å. The Nd1-O1-Nd1^i^ (Symmetry codes: (i) -x+1, -y+1, -z+1) angle is 101.96 (13)°, the resulting Nd···Nd intradimer separation is 4.1259 (4) Å indicates that the metal···metal distances are primarily governed by the nature and mode of the coordination of the bridging groups (Sun *et al.*, 2002). The carboxylate group shows a distortion from the molecular plane; the dihedral angle between the mean-planes of the benzene ring (C2-C7; plane 1) and the carboxlate group (O2/C1/O3^i^) is 7.7 (6)°, and that between the mean-planes of benzene ring (C9-C14; plane 2) and the O1/C8/O4 carboxlate group is 24.4 (5)°. The two carboxylate groups are almost perpendicular to one another with a dihedral angle of 84.0 (7) °, and planes 1 and 2 are inclined to one another by 81.8 (2) ° compared with the corresponding value found in the complex [La_2_(C_7_H_7_NO_2_)_4_Cl_2_(H_2_O)_6_]Cl_4_.2H_2_O [(Benslimane *et al.*, 2011) 80.0 (2)].

In the crystal hydrogen bonds involving the free and the coordinated water molecules, the ammonium group NH_3_ and the Cl atoms build up a three dimensionnal network (Fig. 2, Table 1). There is also slipped π -π stacking interactions between the symetry related C9—C14 phenyl ring (Table 2). Both hydrogen-bonding and π-π interactions combine to stabilize the three-dimensional network.

### Experimental

NdCl_3_(0.25 g, 1mmol) was dissolved in an aqueous solution of NaOH (0.5 M, 25 ml) with constant stirring. 3-aminobenzoic acid (0.14 g, 1 mmol) was added to the mixture and the pH was adjusted to ca. 3 using 4M HCl. The mixture was refluxed at 353K for about 1 h and then cooled to room temperature. Slow evaporation of the solvent at room temperature lead to the formation of prismatic purple crystals of the title compound.

### Refinement

The chloride anion Cl3 is disordered over three sites, Cl3A, Cl3B and Cl3C, which were refined with occupancies of 0.75, 0.15 and 0.10, respectively. The water molecule O6W is also disordered over two positions (O6WA and O6WB), which were refined with occupancy factors 0.72/0.28, so no H-atoms could be reliably defined. All H atoms attached to C and N atoms were fixed geometrically and treated as riding with C—H = 0.93 Å and N—H = 0.89 Å with *U*_iso_(H) = 1.2*U*_eq_(C) or *U*_iso_(H) = 1.5*U*_eq_(N). The H-atoms of the coordinated water molecules were initially refined using distance restraints [O—H = 0.85 (2) Å, and H···H = 1.40 (2) Å] with *U*_iso_(H) = 1.5*U*_eq_(O). However, in the last cycles of refinement, they were treated as riding on their parent O atoms.

### Crystal data

**Table:**

[Nd_2_(C_7_H_7_NO_2_)_4_(H_2_O)_8_]Cl_6_·4H_2_O	*F*(000) = 1260
*M**_r_* = 1265.92	*D*_x_ = 1.784 Mg m^−^^3^
Monoclinic, *P*2_1_/*c*	Mo *K*α radiation, λ = 0.71073 Å
Hall symbol: -P 2ybc	Cell parameters from 7192 reflections
*a* = 12.1717 (1) Å	θ = 1.0–30.0°
*b* = 19.8544 (1) Å	µ = 2.59 mm^−^^1^
*c* = 10.5170 (1) Å	*T* = 293 K
β = 112.018 (1)°	Prism, violet
*V* = 2356.19 (4) Å^3^	0.30 × 0.24 × 0.16 mm
*Z* = 2	

### Data collection

**Table:**

Enraf–Nonius CAD-4 diffractometer	*R*_int_ = 0.031
graphite	θ_max_ = 30.0°, θ_min_ = 1.8°
non–profiled ω/2τ scans	*h* = −15→17
Absorption correction: multi-scan (Blessing, 1997)	*k* = −27→0
*T*_min_ = 0.410, *T*_max_ = 0.444	*l* = −14→0
7192 measured reflections	2 standard reflections every 60 min
6852 independent reflections	intensity decay: 3%
4724 reflections with *I* > 2σ(*I*)	

### Refinement

**Table:**

Refinement on *F*^2^	Primary atom site location: structure-invariant direct methods
Least-squares matrix: full	Secondary atom site location: difference Fourier map
*R*[*F*^2^ > 2σ(*F*^2^)] = 0.041	Hydrogen site location: inferred from neighbouring sites
*wR*(*F*^2^) = 0.096	H-atom parameters constrained
*S* = 1.02	*w* = 1/[σ^2^(*F*_o_^2^) + (0.0201*P*)^2^] where *P* = (*F*_o_^2^ + 2*F*_c_^2^)/3
6852 reflections	(Δ/σ)_max_ = 0.002
300 parameters	Δρ_max_ = 0.81 e Å^−^^3^
0 restraints	Δρ_min_ = −1.15 e Å^−^^3^

### Special details

**Table:**

Geometry. Bond distances, angles etc. have been calculated using the rounded fractional coordinates. All su's are estimated from the variances of the (full) variance-covariance matrix. The cell esds are taken into account in the estimation of distances, angles and torsion angles
Refinement. Refinement of F^2^ against ALL reflections. The weighted R-factor wR and goodness of fit S are based on F^2^, conventional R-factors R are based on F, with F set to zero for negative F^2^. The threshold expression of F^2^ > 2sigma(F^2^) is used only for calculating R-factors(gt) etc. and is not relevant to the choice of reflections for refinement. R-factors based on F^2^ are statistically about twice as large as those based on F, and R- factors based on ALL data will be even larger.

### Fractional atomic coordinates and isotropic or equivalent isotropic displacement parameters (Å^2^)

**Table:**

	*x*	*y*	*z*	*U*_iso_*/*U*_eq_	Occ. (<1)
Nd1	0.67791 (2)	0.47629 (1)	0.58312 (3)	0.0236 (1)	
O1	0.5246 (3)	0.48149 (15)	0.3573 (4)	0.0328 (10)	
O1W	0.8553 (3)	0.53988 (16)	0.7384 (4)	0.0470 (13)	
O2	0.5576 (2)	0.38174 (14)	0.5941 (4)	0.0338 (12)	
O2W	0.7992 (3)	0.51554 (18)	0.4499 (5)	0.0522 (14)	
O3	0.6299 (3)	0.59263 (14)	0.5323 (4)	0.0318 (10)	
O3W	0.7348 (3)	0.37860 (16)	0.4667 (5)	0.0516 (16)	
O4	0.6548 (3)	0.50923 (15)	0.7970 (4)	0.0336 (13)	
O4W	0.8127 (3)	0.40099 (16)	0.7663 (5)	0.0518 (16)	
N1	0.1193 (4)	0.2246 (2)	0.5525 (7)	0.063 (2)	
N2	0.2428 (3)	0.5776 (2)	0.9569 (5)	0.0432 (16)	
C1	0.4479 (4)	0.36950 (19)	0.5480 (5)	0.0264 (14)	
C2	0.4066 (4)	0.30648 (19)	0.5946 (5)	0.0265 (14)	
C3	0.4840 (4)	0.2636 (2)	0.6904 (6)	0.0334 (16)	
C4	0.4435 (5)	0.2104 (2)	0.7436 (6)	0.0422 (18)	
C5	0.3240 (5)	0.1978 (2)	0.7016 (7)	0.0450 (19)	
C6	0.2491 (4)	0.2391 (2)	0.6034 (7)	0.0410 (18)	
C7	0.2861 (4)	0.2928 (2)	0.5493 (6)	0.0354 (16)	
C8	0.5479 (4)	0.5265 (2)	0.7622 (5)	0.0258 (14)	
C9	0.5114 (4)	0.5576 (2)	0.8680 (5)	0.0266 (14)	
C10	0.5955 (4)	0.5888 (2)	0.9817 (6)	0.0315 (14)	
C11	0.5631 (4)	0.6181 (2)	1.0799 (6)	0.0391 (19)	
C12	0.4470 (4)	0.6159 (2)	1.0698 (6)	0.0410 (19)	
C13	0.3648 (4)	0.5835 (2)	0.9597 (6)	0.0332 (14)	
C14	0.3941 (4)	0.5556 (2)	0.8575 (6)	0.0300 (14)	
O5W	0.9028 (5)	0.6248 (3)	0.3390 (7)	0.126 (3)	
Cl1	0.08111 (11)	0.07403 (8)	0.44309 (19)	0.0593 (6)	
Cl2	0.93715 (13)	0.40966 (10)	0.3509 (2)	0.0708 (7)	
Cl3A	0.8142 (3)	0.22254 (14)	0.4682 (4)	0.0718 (12)	0.750
O6WA	0.0280 (8)	0.2247 (5)	0.7815 (11)	0.081 (4)	0.720
Cl3B	0.7983 (15)	0.2369 (6)	0.5423 (16)	0.068 (5)	0.150
Cl3C	0.8035 (14)	0.2679 (7)	0.731 (3)	0.076 (8)	0.100
O6WB	0.042 (2)	0.2654 (13)	0.750 (3)	0.101 (11)	0.280
H01	0.67440	0.58980	0.99080	0.0380*	
H1A	0.09130	0.23580	0.61660	0.0940*	
H1B	0.10720	0.18090	0.53370	0.0940*	
H1C	0.08210	0.24840	0.47680	0.0940*	
H2	0.61980	0.63960	1.15420	0.0470*	
H02	0.42470	0.63590	1.13640	0.0490*	
H2A	0.23900	0.54320	1.00940	0.0650*	
H2B	0.19280	0.57050	0.87110	0.0650*	
H2C	0.22310	0.61540	0.98840	0.0650*	
H3	0.56510	0.27100	0.71920	0.0400*	
H04	0.33630	0.53550	0.78210	0.0360*	
H4	0.49720	0.18260	0.80860	0.0500*	
H5	0.29540	0.16250	0.73860	0.0540*	
H7	0.23170	0.31980	0.48320	0.0420*	
H11	0.92430	0.55420	0.72050	0.0700*	
H12	0.84330	0.56120	0.48200	0.0790*	
H13	0.78720	0.38670	0.42090	0.0770*	
H14	0.79880	0.35900	0.77100	0.0500*	
H21	0.87730	0.53200	0.81700	0.0700*	
H22	0.83660	0.47750	0.44180	0.0790*	
H23	0.74080	0.34060	0.49470	0.0770*	
H24	0.88800	0.41770	0.81750	0.0500*	
H15W	0.94470	0.63530	0.29310	0.1890*	
H25W	0.86390	0.65960	0.34420	0.1890*	

### Atomic displacement parameters (Å^2^)

**Table:**

	*U*^11^	*U*^22^	*U*^33^	*U*^12^	*U*^13^	*U*^23^
Nd1	0.0204 (1)	0.0285 (1)	0.0211 (2)	−0.0019 (1)	0.0066 (1)	−0.0007 (1)
O1	0.0360 (16)	0.0417 (17)	0.019 (2)	−0.0037 (14)	0.0085 (16)	−0.0053 (16)
O1W	0.0292 (16)	0.069 (2)	0.037 (3)	−0.0175 (15)	0.0057 (18)	−0.0001 (19)
O2	0.0273 (15)	0.0316 (16)	0.040 (3)	−0.0036 (12)	0.0097 (16)	0.0021 (15)
O2W	0.064 (2)	0.054 (2)	0.052 (3)	−0.0250 (18)	0.037 (2)	−0.020 (2)
O3	0.0314 (15)	0.0278 (15)	0.031 (2)	−0.0023 (12)	0.0057 (16)	−0.0001 (14)
O3W	0.073 (2)	0.0372 (19)	0.064 (4)	0.0064 (17)	0.048 (3)	0.0026 (19)
O4	0.0321 (16)	0.0421 (18)	0.028 (3)	0.0005 (13)	0.0129 (17)	−0.0040 (15)
O4W	0.0349 (17)	0.047 (2)	0.056 (4)	0.0004 (15)	−0.003 (2)	0.007 (2)
N1	0.047 (3)	0.061 (3)	0.082 (6)	−0.023 (2)	0.026 (3)	0.004 (3)
N2	0.041 (2)	0.055 (3)	0.041 (3)	0.0043 (19)	0.024 (2)	−0.002 (2)
C1	0.035 (2)	0.0224 (19)	0.025 (3)	−0.0056 (16)	0.015 (2)	−0.0054 (18)
C2	0.031 (2)	0.025 (2)	0.024 (3)	−0.0044 (16)	0.011 (2)	−0.0034 (18)
C3	0.037 (2)	0.028 (2)	0.031 (4)	−0.0027 (17)	0.008 (2)	−0.007 (2)
C4	0.059 (3)	0.028 (2)	0.034 (4)	0.000 (2)	0.011 (3)	0.003 (2)
C5	0.059 (3)	0.035 (3)	0.045 (4)	−0.010 (2)	0.024 (3)	0.006 (2)
C6	0.037 (2)	0.041 (3)	0.050 (4)	−0.012 (2)	0.022 (3)	−0.008 (3)
C7	0.032 (2)	0.032 (2)	0.040 (4)	−0.0040 (18)	0.011 (2)	0.000 (2)
C8	0.032 (2)	0.0269 (19)	0.019 (3)	−0.0076 (18)	0.010 (2)	−0.005 (2)
C9	0.036 (2)	0.026 (2)	0.017 (3)	0.0001 (17)	0.009 (2)	0.0003 (18)
C10	0.032 (2)	0.037 (2)	0.025 (3)	0.0012 (18)	0.010 (2)	−0.004 (2)
C11	0.045 (3)	0.045 (3)	0.026 (4)	−0.006 (2)	0.012 (3)	−0.016 (2)
C12	0.051 (3)	0.045 (3)	0.028 (4)	0.007 (2)	0.016 (3)	−0.010 (2)
C13	0.034 (2)	0.037 (2)	0.031 (3)	0.0070 (19)	0.015 (2)	0.003 (2)
C14	0.034 (2)	0.033 (2)	0.021 (3)	−0.0010 (18)	0.008 (2)	−0.001 (2)
O5W	0.140 (5)	0.124 (5)	0.118 (7)	0.037 (4)	0.052 (5)	0.024 (5)
Cl1	0.0401 (7)	0.0667 (9)	0.0549 (13)	−0.0080 (6)	−0.0009 (8)	0.0083 (8)
Cl2	0.0441 (8)	0.1218 (14)	0.0541 (14)	−0.0135 (8)	0.0270 (9)	−0.0161 (11)
Cl3A	0.0611 (13)	0.0457 (14)	0.099 (3)	−0.0090 (11)	0.0190 (19)	0.0111 (15)
O6WA	0.058 (4)	0.129 (8)	0.060 (7)	0.000 (5)	0.027 (5)	−0.006 (6)
Cl3B	0.093 (10)	0.023 (5)	0.056 (11)	0.000 (5)	−0.008 (9)	0.007 (5)
Cl3C	0.071 (10)	0.042 (8)	0.12 (2)	0.013 (7)	0.042 (12)	0.001 (9)
O6WB	0.086 (15)	0.15 (2)	0.07 (2)	0.007 (16)	0.034 (14)	0.029 (18)

### Geometric parameters (Å, °)

**Table:**

Nd1—O1	2.411 (4)	N1—H1A	0.8900
Nd1—O1W	2.506 (4)	N2—H2A	0.8900
Nd1—O2	2.410 (3)	N2—H2B	0.8900
Nd1—O2W	2.510 (4)	N2—H2C	0.8900
Nd1—O3	2.394 (3)	C1—C2	1.498 (6)
Nd1—O3W	2.525 (4)	C2—C3	1.384 (7)
Nd1—O4	2.458 (4)	C2—C7	1.389 (7)
Nd1—O4W	2.504 (4)	C3—C4	1.371 (7)
Nd1—O1^i^	2.886 (4)	C4—C5	1.375 (9)
O1—C8^i^	1.246 (6)	C5—C6	1.365 (8)
O2—C1	1.262 (6)	C6—C7	1.362 (7)
O3—C1^i^	1.255 (6)	C8—C9	1.479 (7)
O4—C8	1.260 (6)	C9—C14	1.391 (8)
O1W—H11	0.9700	C9—C10	1.394 (7)
O1W—H21	0.7800	C10—C11	1.366 (8)
O2W—H12	1.0400	C11—C12	1.378 (8)
O2W—H22	0.9000	C12—C13	1.374 (8)
O3W—H23	0.8000	C13—C14	1.370 (8)
O3W—H13	0.9500	C3—H3	0.9300
O4W—H14	0.8600	C4—H4	0.9300
O4W—H24	0.9300	C5—H5	0.9300
O5W—H25W	0.8500	C7—H7	0.9300
O5W—H15W	0.8500	C10—H01	0.9300
N1—C6	1.494 (8)	C11—H2	0.9300
N2—C13	1.479 (7)	C12—H02	0.9300
N1—H1C	0.8900	C14—H04	0.9300
N1—H1B	0.8900		
			
O1—Nd1—O1W	141.27 (11)	C6—N1—H1C	109.00
O1—Nd1—O2	79.67 (12)	C6—N1—H1A	110.00
O1—Nd1—O2W	80.61 (14)	C6—N1—H1B	109.00
O1—Nd1—O3	72.82 (12)	H1A—N1—H1B	109.00
O1—Nd1—O3W	78.87 (13)	H1B—N1—H1C	109.00
O1—Nd1—O4	125.33 (13)	H1A—N1—H1C	110.00
O1—Nd1—O4W	145.66 (11)	H2B—N2—H2C	110.00
O1—Nd1—O1^i^	78.04 (12)	H2A—N2—H2B	109.00
O1W—Nd1—O2	138.75 (12)	C13—N2—H2A	109.00
O1W—Nd1—O2W	70.36 (14)	C13—N2—H2B	109.00
O1W—Nd1—O3	74.80 (12)	H2A—N2—H2C	109.00
O1W—Nd1—O3W	112.16 (13)	C13—N2—H2C	110.00
O1W—Nd1—O4	68.64 (13)	O2—C1—C2	118.2 (4)
O1W—Nd1—O4W	69.11 (12)	O2—C1—O3^i^	124.5 (4)
O1^i^—Nd1—O1W	108.03 (11)	O3^i^—C1—C2	117.4 (4)
O2—Nd1—O2W	139.79 (12)	C3—C2—C7	118.2 (4)
O2—Nd1—O3	131.34 (12)	C1—C2—C3	122.1 (5)
O2—Nd1—O3W	72.96 (12)	C1—C2—C7	119.5 (4)
O2—Nd1—O4	83.30 (12)	C2—C3—C4	121.4 (5)
O2—Nd1—O4W	74.50 (12)	C3—C4—C5	120.5 (5)
O1^i^—Nd1—O2	68.41 (9)	C4—C5—C6	117.4 (5)
O2W—Nd1—O3	73.81 (13)	N1—C6—C5	118.2 (5)
O2W—Nd1—O3W	69.04 (12)	N1—C6—C7	118.2 (5)
O2W—Nd1—O4	136.16 (13)	C5—C6—C7	123.7 (5)
O2W—Nd1—O4W	105.19 (14)	C2—C7—C6	118.8 (5)
O1^i^—Nd1—O2W	139.46 (11)	O1^i^—C8—O4	121.5 (5)
O3—Nd1—O3W	136.17 (14)	O1^i^—C8—C9	120.9 (5)
O3—Nd1—O4	81.11 (12)	O4—C8—C9	117.6 (4)
O3—Nd1—O4W	141.52 (13)	C10—C9—C14	118.9 (5)
O1^i^—Nd1—O3	67.12 (11)	C8—C9—C14	121.2 (4)
O3W—Nd1—O4	142.60 (12)	C8—C9—C10	120.0 (5)
O3W—Nd1—O4W	72.23 (14)	C9—C10—C11	120.7 (5)
O1^i^—Nd1—O3W	137.75 (11)	C10—C11—C12	120.5 (5)
O4—Nd1—O4W	73.83 (13)	C11—C12—C13	118.8 (5)
O1^i^—Nd1—O4	47.46 (12)	N2—C13—C14	120.5 (5)
O1^i^—Nd1—O4W	111.90 (12)	C12—C13—C14	122.0 (5)
Nd1—O1—Nd1^i^	101.96 (13)	N2—C13—C12	117.6 (5)
Nd1—O1—C8^i^	169.3 (3)	C9—C14—C13	119.2 (5)
Nd1^i^—O1—C8^i^	85.2 (3)	C2—C3—H3	119.00
Nd1—O2—C1	134.9 (3)	C4—C3—H3	119.00
Nd1—O3—C1^i^	142.0 (3)	C5—C4—H4	120.00
Nd1—O4—C8	105.5 (3)	C3—C4—H4	120.00
H11—O1W—H21	107.00	C4—C5—H5	121.00
Nd1—O1W—H11	128.00	C6—C5—H5	121.00
Nd1—O1W—H21	117.00	C6—C7—H7	121.00
H12—O2W—H22	123.00	C2—C7—H7	121.00
Nd1—O2W—H22	102.00	C9—C10—H01	120.00
Nd1—O2W—H12	115.00	C11—C10—H01	120.00
Nd1—O3W—H23	123.00	C12—C11—H2	120.00
H13—O3W—H23	111.00	C10—C11—H2	120.00
Nd1—O3W—H13	118.00	C11—C12—H02	121.00
Nd1—O4W—H24	117.00	C13—C12—H02	121.00
H14—O4W—H24	119.00	C9—C14—H04	120.00
Nd1—O4W—H14	123.00	C13—C14—H04	120.00
H15W—O5W—H25W	108.00		
			
O1W—Nd1—O1—Nd1^i^	−103.98 (19)	O4W—Nd1—O4—C8	−146.0 (3)
O2—Nd1—O1—Nd1^i^	69.90 (11)	O1^i^—Nd1—O4—C8	−3.6 (2)
O2W—Nd1—O1—Nd1^i^	−145.32 (12)	Nd1—O1^i^—C8—C9	173.3 (4)
O3—Nd1—O1—Nd1^i^	−69.49 (12)	Nd1—O1^i^—C8—O4	−5.9 (4)
O3W—Nd1—O1—Nd1^i^	144.37 (13)	Nd1—O2—C1—O3^i^	6.6 (8)
O4—Nd1—O1—Nd1^i^	−4.17 (16)	Nd1—O2—C1—C2	−171.7 (3)
O4W—Nd1—O1—Nd1^i^	111.5 (2)	Nd1^i^—O3^i^—C1—C2	142.4 (4)
O1^i^—Nd1—O1—Nd1^i^	0.00 (9)	Nd1^i^—O3^i^—C1—O2	−35.9 (9)
O1^i^—Nd1^i^—O1—Nd1	0.00 (11)	Nd1—O4—C8—O1^i^	7.1 (5)
O1W^i^—Nd1^i^—O1—Nd1	−140.33 (11)	Nd1—O4—C8—C9	−172.1 (3)
O2^i^—Nd1^i^—O1—Nd1	83.50 (13)	O3^i^—C1—C2—C7	−0.6 (7)
O2W^i^—Nd1^i^—O1—Nd1	−59.7 (2)	O2—C1—C2—C7	177.8 (5)
O3^i^—Nd1^i^—O1—Nd1	−76.21 (13)	O2—C1—C2—C3	3.0 (7)
O3W^i^—Nd1^i^—O1—Nd1	58.2 (2)	O3^i^—C1—C2—C3	−175.4 (5)
O4^i^—Nd1^i^—O1—Nd1	−175.39 (17)	C1—C2—C3—C4	172.4 (5)
O4W^i^—Nd1^i^—O1—Nd1	145.54 (12)	C1—C2—C7—C6	−173.3 (5)
O1—Nd1—O2—C1	−36.4 (4)	C7—C2—C3—C4	−2.4 (8)
O1W—Nd1—O2—C1	137.8 (4)	C3—C2—C7—C6	1.7 (7)
O2W—Nd1—O2—C1	−98.2 (5)	C2—C3—C4—C5	0.7 (8)
O3—Nd1—O2—C1	19.5 (5)	C3—C4—C5—C6	1.6 (8)
O3W—Nd1—O2—C1	−117.8 (5)	C4—C5—C6—N1	177.7 (5)
O4—Nd1—O2—C1	91.4 (4)	C4—C5—C6—C7	−2.4 (9)
O4W—Nd1—O2—C1	166.5 (5)	C5—C6—C7—C2	0.7 (9)
O1^i^—Nd1—O2—C1	44.7 (4)	N1—C6—C7—C2	−179.3 (5)
O1—Nd1—O3—C1^i^	15.9 (5)	O4—C8—C9—C10	23.0 (6)
O1W—Nd1—O3—C1^i^	174.4 (6)	O1^i^—C8—C9—C14	24.7 (6)
O2—Nd1—O3—C1^i^	−42.6 (6)	O4—C8—C9—C14	−156.1 (4)
O2W—Nd1—O3—C1^i^	100.9 (6)	O1^i^—C8—C9—C10	−156.2 (4)
O3W—Nd1—O3—C1^i^	68.0 (6)	C8—C9—C10—C11	179.6 (4)
O4—Nd1—O3—C1^i^	−115.5 (6)	C14—C9—C10—C11	−1.3 (7)
O4W—Nd1—O3—C1^i^	−165.0 (5)	C8—C9—C14—C13	178.3 (4)
O1^i^—Nd1—O3—C1^i^	−68.1 (6)	C10—C9—C14—C13	−0.8 (6)
O1—Nd1—O4—C8	2.0 (3)	C9—C10—C11—C12	1.5 (7)
O1W—Nd1—O4—C8	140.5 (3)	C10—C11—C12—C13	0.3 (7)
O2—Nd1—O4—C8	−70.3 (3)	C11—C12—C13—N2	175.6 (4)
O2W—Nd1—O4—C8	118.7 (3)	C11—C12—C13—C14	−2.5 (7)
O3—Nd1—O4—C8	63.5 (3)	N2—C13—C14—C9	−175.3 (4)
O3W—Nd1—O4—C8	−120.5 (3)	C12—C13—C14—C9	2.7 (7)

Symmetry codes: (i) −*x*+1, −*y*+1, −*z*+1.

### Hydrogen-bond geometry (Å, °)

**Table:**

*D*—H···*A*	*D*—H	H···*A*	*D*···*A*	*D*—H···*A*
N1—H1A···O6WA	0.89	2.16	3.007 (12)	160
N1—H1B···Cl1	0.89	2.30	3.174 (5)	168
N1—H1C···O6WA^ii^	0.89	1.98	2.828 (12)	159
N2—H2A···O4^iii^	0.89	2.22	2.967 (6)	141
N2—H2B···Cl2^i^	0.89	2.31	3.166 (5)	161
N2—H2C···Cl3A^iv^	0.89	2.26	3.129 (5)	167
O1W—H11···Cl2^v^	0.97	2.21	3.170 (4)	172
O2W—H12···O5W	1.04	2.28	2.960 (7)	121
O2W—H12···Cl2^v^	1.04	2.65	3.443 (5)	132
O3W—H13···Cl2	0.95	2.26	3.190 (5)	169
O4W—H14···Cl3A^vi^	0.86	2.58	3.240 (5)	134
O5W—H15W···Cl1^vii^	0.85	2.68	3.208 (7)	122
O1W—H21···Cl1^iv^	0.78	2.52	3.215 (4)	148
O2W—H22···Cl2	0.90	2.26	3.104 (4)	156
O3W—H23···Cl3A	0.80	2.56	3.244 (4)	144
O4W—H24···Cl1^viii^	0.93	2.23	3.134 (5)	163
O5W—H25W···N1^i^	0.85	2.52	3.247 (8)	144
C10—H01···Cl1^iv^	0.93	2.81	3.724 (6)	169
C14—H04···O2W^i^	0.93	2.59	3.504 (7)	170

Symmetry codes: (ii) *x*, −*y*+1/2, *z*−1/2; (iii) −*x*+1, −*y*+1, −*z*+2; (i) −*x*+1, −*y*+1, −*z*+1; (iv) −*x*+1, *y*+1/2, −*z*+3/2; (v) −*x*+2, −*y*+1, −*z*+1; (vi) *x*, −*y*+1/2, *z*+1/2; (vii) −*x*+1, *y*+1/2, −*z*+1/2; (viii) *x*+1, −*y*+1/2, *z*+1/2.

### Table 2 Table 2 π-π stacking interactions (Å)

Cg1 is the centroid of the C9—C14 ring.

**Table:**

CgI	CgJ	CgI···CgJ^a^	CgI···P(J)^b^	CgJ···P(I)^c^	Slippage
Cg1	Cg1^ii^	3.499 (2)	3.2720 (18)	3.2721 (18)	1.240

Symmetry code: (ii) 1-x,1-y,2-z. Notes: (a) Distance between centroids; (b) Perpendicular distance of CgI on ring plan J; (c) Perpendicular distance of CgJ on ring plan I. Slippage = vertical displacement between ring centroids.
